# Weighted Structured Sparse Reconstruction-Based Lamb Wave Imaging Exploiting Multipath Edge Reflections in an Isotropic Plate

**DOI:** 10.3390/s20123502

**Published:** 2020-06-21

**Authors:** Caibin Xu, Zhibo Yang, Mingxi Deng

**Affiliations:** 1 College of Aerospace Engineering, Chongqing University, Chongqing 400044, China; caibinxu0839@cqu.edu.cn; 2 School of Mechanical Engineering, Xi’an Jiaotong University, Xi’an 710049, China; phdapple@mail.xjtu.edu.cn

**Keywords:** lamb wave, structured sparse reconstruction, imaging algorithm, defect detection, structural health monitoring

## Abstract

Lamb wave-based structural health monitoring techniques have the ability to scan a large area with relatively few sensors. Lamb wave imaging is a signal processing strategy that generates an image for locating scatterers according to the received Lamb waves. This paper presents a Lamb wave imaging method, which is formulated as a weighted structured sparse reconstruction problem. A dictionary is constructed by an analytical Lamb wave scattering model and an edge reflection prediction technique, which is used to decompose the experimental scattering signals under the constraint of weighted structured sparsity. The weights are generated from the correlation coefficients between the scattering signals and the predicted ones. Simulation and experimental results from an aluminum plate verify the effectiveness of the present method, which can generate images with sparse pixel values even with very limited number of sensors.

## 1. Introduction

Lamb waves are ultrasonic guided waves in thin plates and have received great attention from researchers in the field of structural health monitoring (SHM) and nondestructive evaluation (NDE) [1,2,3]. Lamb waves are an attractive tool for SHM and NDE because of their ability to achieve large-scale and long-distance detection as well as their sensitivity to several types of damage such as crack, delamination and corrosion [4,5,6,7]. A typical Lamb wave-based SHM or NDE system usually consists of a sensor array to receive the responses to a specific excitation. The structural health condition is evaluated based on the current measured data and the baseline data which is recorded when the structure is damage-free. Usually, the evaluation is more precise if more data collected from different receivers is available, but it also means a higher cost at the same time.

Flaws or damage in structures can be seen as scatterers that scatter the incoming waves in different directions. Then a variety of strategies can be developed to process the scattering signals to detect and locate the flaws or damage. Guided wave imaging method is one of those strategies that can generate an image indicating the number and locations of damage. One of such well-known methods is the delay-and-sum imaging method developed by Wang et al. [8]. This method uses the wave velocity and the time-of-flight to locate the positions of damage. Another imaging method, the minimum variance distortionless response imaging method [9], is a variation of the delay-and-sum, in which adaptive weights are introduced to improve imaging performance. Zhao et al. developed an imaging method known as the reconstruction algorithm for probabilistic inspection of defects (RAPID) [10], which can be included in the category of statistical imaging methods. The RAPID uses the signal difference coefficient between the current measured data and the baseline data as damage index (DI). Since the RAPID does not require the wave velocity for imaging, it is suitable for complex structures such as wind turbine blades, aircraft wings and variable thickness plates. Other statistical imaging methods may use the difference of the fractal dimension [11] and the signal entropy [12] between two signals to be DIs. However, the above imaging methods have limited imaging performance unless dense transmitter-receiver pairs are available.

Dictionary-based approaches are another kind of imaging methods that usually use an analytical or finite element-based guided wave propagation models to generate lots of scattering signals as the atoms of a dictionary. Alguri et al. used guided wave data collected from surrogate structures to learn a dictionary and then reconstructed the wave field of the structure under test [13]. Levine et al. sparsely decomposed the scattering signals in a dictionary generated by an analytical Lamb wave propagation model, which formulated the Lamb wave imaging as a sparse or block-sparse reconstruction problem [14,15]. With the use of more prior knowledge, super-resolution imaging can be achieved by introducing the sparse reconstruction theory. Hua et al. presented a sparse reconstruction imaging for Lamb wave simultaneous excitation system based on a dictionary [16]. Golato et al. constructed a dictionary composed of multimodal scattering Lamb waves and solved a sparse reconstruction problem to achieve Lamb wave imaging [17]. However, the above approaches did not consider the edge reflections in the process of dictionary construction. Zuo et al. used the model-based predicted signal and the experimental scattering signal to compute the covariance matrix and then used the two-dimensional MUSIC algorithm for Lamb wave imaging in composite laminates [18]. However, this method is only suitable for one dimensional uniform linear array.

In this paper, a weighted structured sparse reconstruction-based Lamb wave imaging method is presented. Lamb wave imaging is formulated as a weighted structured sparse reconstruction problem using a model-based Lamb wave dictionary. In the construction of this dictionary, not only the scattering signal from the excitation source to a potential scatterer to the receiver but also the edge reflections are considered and incorporated. The scattering signals are preprocessed by a model-based sparse decomposition approach so as to reconstruct the scattering signals with desired number of wave packets. This processing can remove some wave packets in the scattering signals and improve the signal-to-noise ratio. The correlation coefficients between the experimental scattering signals and predicted signals are used to construct the weights of the weighted structured sparse reconstruction model.

The rest of the paper is organized as follows. In Section 2, background of the Lamb wave scattering model and the prediction of edge reflections are given. In Section 3, the details of the methodology for Lamb wave imaging is presented. In Section 4 and Section 5, simulation and experimental studies are implemented to validate the present method. Finally, conclusions are drawn in Section 6.

## 2. Background

### 2.1. Lamb Wave Scattering Model

Lamb waves are multimodal and dispersive. A thin plate supports at least two Lamb wave modes at any frequencies. Under the cutoff frequency, only the fundamental A0 and S0 modes will appear in the plate. In addition, a desired single fundamental Lamb wave mode can be transmitted by means of dual wafers energized in-phase/out-of-phase or wavelength turning techniques [19,20]. Due to the dispersion, a Lamb wave packet will spread out in time and space as it propagates. In an infinite thin plate, the single-mode Lamb wave response of a receiver d away from a point-like source to an excitation S(ω) can be expressed as [21]

(1) $$u(t)=\sqrt{\frac{d_{0}}{d}}\frac{1}{2\pi }\int_{-\infty }^{+\infty }S(\omega )e^{j\omega t}e^{-jk(\omega )d}d\omega$$

where u(t) is the response signal in the time domain, $d_{0}$ is an reference distance, t is the time, ω is the angular frequency, S(ω) is the excitation signal in the frequency domain, k(ω) is the wavenumber of the specific Lamb wave mode, and j is the imaginary unit. The frequency form of Equation (1) can be written as

(2) $$U(\omega )=\sqrt{\frac{d_{0}}{d}}S(\omega )e^{-jk(\omega )d}$$

where U(ω) is the frequency form of u(t).

Suppose there is a scatterer in the plate, and the distance from the excitation source to the scatterer to the receiver is $d_{s}$, then the scattering signal $Y_{s}(\omega )$ in the frequency domain can be expressed as

(3) $$Y_{s}(\omega )=\alpha (\omega )\sqrt{\frac{d_{0}}{d_{s}}}S(\omega )e^{-jk(\omega )d_{s}}$$

where the frequency dependent parameter α(ω) is the corresponding scattering coefficient.

In Lamb wave-based SHM, a windowed tone burst centered at a specific frequency is usually selected to be the excitation signal to suppress the dispersion. In such situation, the excitation is a narrowband signal, then the frequency dependent scattering coefficient α(ω) in Equation (3) can be approximated as the value $\alpha (\omega_{c})$. When there are multiple scatterers in the plate, the corresponding scattering signal $Y_{s}(\omega )$ can be approximately expressed as a linear superposition of each single scatterer case without considering the scattering between any two scatterers

(4) $$Y_{s}(\omega )=\sum_{i}\alpha_{i}(\omega )\sqrt{\frac{d_{0}}{d_{s}^{i}}}S(\omega )e^{-jk(\omega )d_{s}^{i}}$$

where $\alpha_{i}(\omega )$ is the scattering coefficient corresponding to the *i*th scatterer, and $d_{s}^{i}$ is the distance from the excitation source to the *i*th scatterer to the receiver.

### 2.2. Prediction of Edge Reflections

In practice, the plate-like structures under test or monitored are all of limited size. Therefore, the recorded Lamb waves by the receiver contains not only the direct scattering signal (direct arrival), but also the edge reflections related to the scatterer. Compared with the direct arrival, the edge reflections arrives later, containing information of the scatterer, and can be incorporated into imaging algorithms to improve imaging performance. The direct arrivals between any two points on the plate can be predicted by Equation (3), and the scatterering signal from the transmitter to the scatter to the receiver can also be predicted by considering the scatterer as a point-like secondary acoustic source if the location of the scatterer is known. The edge reflections also can be predicted by Equation (4) if the traveling distance $d_{s}^{i}$ of each edge reflection is known.

**Figure 1:**
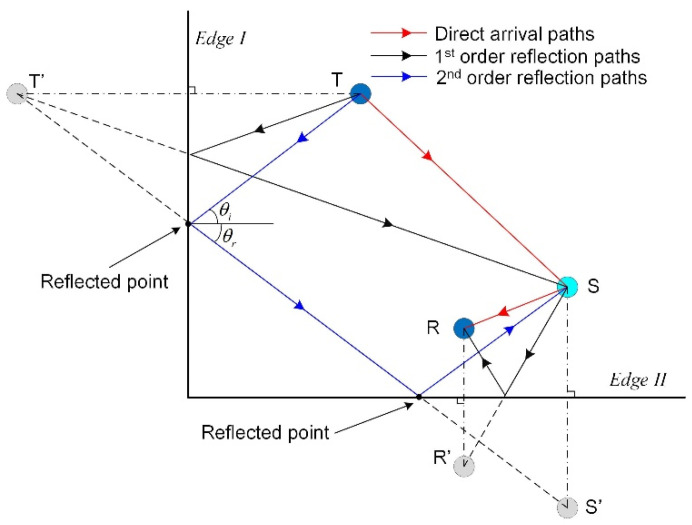
Schematic diagram of the traveling paths of some scattering wave packets from the transmitter to the scatterer to the receiver. The symbol “T” denotes the transmitter, “S” denotes the scatterer, and “R” denotes the receiver. “$T^{\prime }$”, “$R^{\prime }$”, and “$S^{\prime }$” denote the corresponding mirror points of “T”, “S” and “R”.

The ray tracking technique [22] is a geometric algorithm that can be used to calculate the traveling paths of edge reflections. There are lots of traveling paths for an acoustic wave from a source to a specific point, including direct arrival paths and edge reflection paths, as shown in Figure 1. An acoustic wave can reflect at the edges of a structure one or several times, and can be recorded by a receiver finally. Figure 1 shows two 1st order (the number of reflections between two points is equal to 1) and one 2nd order (the number of reflections between two points is equal to 2) reflection paths. The angle of incident wave and the angle of reflected wave at an edge satisfy the Snell’s law

(5) $$k_{i}\sin(\theta_{i})=k_{r}\sin(\theta_{r})$$

where $k_{i}$, $k_{r}$ are the wavenumbers of the incident and reflected waves, and $\theta_{i}$, $\theta_{r}$ are the angles of the incident and reflected waves. If the mode conversion of edge reflections is not considered, $k_{i}$ will be equal to $k_{r}$. Accordingly, the angles of the incident and reflected waves are the same, i.e., $\theta_{i}=\theta_{r}$. In this paper, the mode conversion is not considered so that the edge reflections can be predicted by the following steps:

- Step 1: Calculate the corresponding positions of mirror points for the transmitter (T), scatterer (S), and receiver (R) by considering an edge of the structure as a mirror. For example, the point T’ is the mirror point for the transmitter T corresponding to the mirror, “*Edge I*”, as show in Figure 1.
- Step 2: Calculate the position of each reflected point. The intersection of an edge and the line between a source or its mirror point to a received point or its mirror point is a reflected point.
- Step 3: Calculate each order reflection paths by connecting the source, the corresponding reflected point, and the receiver in turn. Please note that there can be multiple reflected points for a reflection path. For example, the 2nd order reflection path in Figure 1 has two reflected points.
- Step 4: Calculate the length of each path and substitute the lengths into Equation (4). The edge reflections can be predicted by Equation (4) when the scattering coefficients are all determined.

Given the positions of the transmitter, the scatterer, and the receiver, the desired paths of scattering wave packets can be calculated by the ray tracking technique so that the scattering wave packets can be predicted by the four steps above. In application, the scattering signal is obtained by subtracting the “health signal” or “baseline signal” recorded at damage-free state from the measured signal. As the scattering signals obtained from experiments contains direct arrivals as well as edge reflections and those wave packets may also overlap in the time domain, it is not easy to separate those wave packets, which means that the existence of edge reflections in scattering signal is inevitable. Therefore, the predicted scattering signal including edge reflections can better match the scattering signal obtained from experiments, which is useful in the improvement of imaging performance for model-based Lamb wave imaging methods.

## 3. Methodology

### 3.1. Scattering Signal Processing

The scattering signals obtained from experiments can be represented by several predicted scattering wave packets. A scattering signal $y_{s}(t)$ obtained from experiments under the narrowband excitation can be expressed as [3,14]

(6) $$y_{s}(t)={ℱ}^{-1}\left\{\sum_{i=1}^{P}\alpha_{i}\sqrt{\frac{d_{0}}{d_{s}^{i}}}S(\omega )e^{-jk(\omega )d_{s}^{i}}\right\}+n(t)$$

where ${ℱ}^{-1}\left\{\cdot \right\}$ represents the inverse Fourier transform, P is the number of scattering wave packets including direct arrivals and edge reflections, n(t) is noise. Suppose the discrete form of $y_{s}(t)$ is a column vector $y_{s}\in {\mathbb{R}}^{N}$, then $y_{s}$ can be expressed as a linear weighted combination of the atoms in a 2-D dictionary Φ [3]

(7) $$y_{s}=\Phi x+n$$

where $\Phi \in {\mathbb{R}}^{N\times M_{1}}$ ($M_{1}\gg P$) is a matrix, $x\in {\mathbb{R}}^{M_{1}}$ is a coefficient column vector, and $n\in {\mathbb{R}}^{N}$ is a noise term. The *i*th column of dictionary Φ, $a_{i}$, is determined by

(8) $$a_{i}={ℱ}^{-1}\left\{\sqrt{\frac{d_{0}}{d^{i}}}S(\omega )e^{-jk(\omega )d^{i}}\right\}$$

where $d^{i}$ is a user-specified traveling distance corresponding to the *i*th atom $a_{i}$.

Equation (7) is an ill-posed problem and it will have infinite solutions if there is no prior information about x. If the number of atoms, M, is large enough and the traveling distances $d_{s}^{i}$ ($1\leq i\leq P,\;i\in {\mathbb{N}}^{+}$) are totally covered by the given traveling distances $d^{i}$ ($1\leq i\leq M_{1},\;i\in {\mathbb{N}}^{+}$), the scattering signal $y_{s}$ can be sparsely decomposed in the dictionary Φ by solving the following sparse decomposition problem

(9) $$\min\;\frac{1}{2}\;{\left||y_{s}-\Phi x|\right|}_{2}^{2}+\lambda {\left||x|\right|}_{1}$$

where λ>0 is a regularization parameter, which balances the sparsity ($\ell_{1}$-norm) and fidelity ($\ell_{2}$-norm) and is related to the variance of noise. In addition, x is a sparse coefficient vector and its *i*th element $x_{i}$ is the scattering coefficient corresponding to the *i*th atom $a_{i}$ whose traveling distance is $d^{i}$. Every scattering wave packet in $y_{s}$ with a unique traveling distance can be recovered using the solution of Equation (9) and the dictionary Φ. So a new scattering signal ${y^{\prime }}_{s}$ composed of the $P^{\prime }\;(P^{\prime }\leq P)$ scattering wave packets corresponding to the first $P^{\prime }$ smallest traveling distances can be reconstructed by

(10) $${y^{\prime }}_{s}=\Phi {\hat{x}}^{\prime }$$

where ${\hat{x}}^{\prime }$ contains $P^{\prime }$ scattering coefficients corresponding to the first $P^{\prime }$ smallest traveling distances. Through signal sparse decomposition and reconstruction, the number of scattering wave packets in the new scattering signal ${y^{\prime }}_{s}$ can be user-specified so that the predicted scattering signal with the same number of wave packets can better match ${y^{\prime }}_{s}$.

### 3.2. Lamb Wave Imaging Formulated as A Weighted Structured Sparse Reconstruction Problem

Suppose that there is a total of L unique measured signals recorded from the sensor network in the Lamb wave-based SHM system. So L scattering signals are obtained by subtracting the corresponding L “baseline signals” from the L measured signals. In addition, L new scattering signals labeled as ${y^{\prime }}_{1s}$, ${y^{\prime }}_{2s}$, …, ${y^{\prime }}_{Ls}$, can be calculated by Equations (9) and (10), and can be used for imaging. The *i*th new scattering signal ${y^{\prime }}_{is}$ corresponds to the *i*th “transmitter-receiver’’ pair, which is composed of a transmitter and a receiver.

For imaging, the interested area in the monitored structure is discretized into $M_{2}$ grids, and each grid is regarded as a potential scattering source. Usually, the number of grids within damaged area is far less than that within damage-free area. For the *i*th “transmitter-receiver’’ pair, the corresponding *i*th new scattering signal ${y^{\prime }}_{is}$ can be decomposed in a 2-D dictionary $\Psi_{i}\in {\mathbb{R}}^{N\times M_{2}}(M_{2}\gg N,\;1\leq i\leq L,\;i\in {\mathbb{N}}^{+})$

(11) $${y^{\prime }}_{is}=\Psi_{i}z_{i}+e_{i}$$

where $e_{i}\in {\mathbb{R}}^{N}$ is a noise term, $z_{i}=[z_{1i},\;z_{2i},\;\ldots ,\;z_{mi},\;\ldots ,\;z_{M_{2}i}{]}^{T}\in {\mathbb{R}}^{M_{2}}$$\left(1\leq i\leq L,\;1\leq m\leq M_{2},\;m\in {\mathbb{N}}^{+}\right)$ is a coefficient column vector, and $\Psi_{i}=[{a^{\prime }}_{1i},\;{a^{\prime }}_{2i},\;\ldots ,\;{a^{\prime }}_{mi},\;\ldots ,{a^{\prime }}_{M_{2}i}]$ is a matrix whose *m*th atom (column) ${a^{\prime }}_{mi}\in {\mathbb{R}}^{N}$ is defined as

(12) $${a^{\prime }}_{mi}={ℱ}^{-1}\left\{\sum_{p=1}^{P^{\prime }}\sqrt{\frac{d_{0}}{d_{p}^{mi}}}S(\omega )e^{-jk(\omega )d_{p}^{mi}}\right\}$$

where $d_{p}^{mi}$ is the traveling distance corresponding to the *p*th scattering wave packet calculated by the ray tracking technique when the scatterer is located at the *m*th grid. In Equation (11), the coefficient $z_{mi}$ in the vector $z_{i}$ quantifies the contribution of the atom ${a^{\prime }}_{mi}$ to the signal ${y^{\prime }}_{is}$. So the probability of damage in the *m*th grid can be characterized using the absolute value of $z_{mi}$.

As there are a total of L new scattering signals, the same operation like Equation (11) can be applied to each signal. So one can obtain the following L equations

(13) $$\left\{ \begin{array}{l} {y^{\prime }}_{1s}=\Psi_{1}z_{1}+e_{1}\\ {y^{\prime }}_{2s}=\Psi_{2}z_{2}+e_{2}\\ \;\vdots \\ {y^{\prime }}_{Ls}=\Psi_{L}z_{L}+e_{L} \end{array}\right.$$

The L equations above can be formulated as

(14) $$\overset{y}{\overbrace{\left[\begin{array}{c} {y^{\prime }}_{1s}\\ {y^{\prime }}_{2s}\\ \vdots \\ {y^{\prime }}_{Ls} \end{array}\right]}}=\overset{\Psi }{\overbrace{\left[\begin{array}{cccc} \Psi_{1} & 0 & \cdots & 0\\ 0 & \Psi_{2} & \cdots & \vdots \\ \vdots & \vdots & \ddots & 0\\ 0 & 0 & \cdots & \Psi_{L} \end{array}\right]}}\overset{z}{\overbrace{\left[\begin{array}{l} z_{1}\\ z_{2}\\ \vdots \\ z_{L} \end{array}\right]}}+\overset{e}{\overbrace{\left[\begin{array}{l} e_{1}\\ e_{2}\\ \vdots \\ e_{L} \end{array}\right]}}$$

where $y\in {\mathbb{R}}^{LN}$ is a column vector including all the new scattering signals and can be calculated from experimental signals, $\Psi \in {\mathbb{R}}^{LN\times LM_{2}}$ is a 2-D dictionary (matrix) constructed using the Lamb wave scattering model and the ray tracking technique, and $z\in {\mathbb{R}}^{LM_{2}}$ is an unknown column vector whose elements characterize the probability of damage for the total of $M_{2}$ grids. 

If there is a scatterer located in the *m*th grid, the *m*th elements of $z_{1}$, $z_{2}$, …, $z_{L}$ all should be nonzero. Or rather, the vectors $z_{1}$, $z_{2}$, …, and $z_{L}$ should have the same sparse structure, i.e., the nonzero elements of $z_{1}$, $z_{2}$, …, and $z_{L}$ should locate at the same positions. Therefore, the vector z has the characteristic of structured sparsity. A solution of Equation (14) can be obtained by solving the following weighted structured sparse reconstruction problem [23]

(15) $$\min\sum_{m=1}^{M_{2}}w_{m}{\left||\phi (m,z)|\right|}_{2}\;s.t.\;{\left||y-\Psi z|\right|}_{2}^{2}\leq \sigma^{2}$$

where $\phi (m,z)={\left[z_{m1},z_{m2},\ldots ,z_{mL}\right]}^{T}\in {\mathbb{R}}^{L}$ is a vector composed of the *m*th elements of $z_{1}$, $z_{2}$, …, $z_{L}$, $\sigma^{2}$ is a regularization parameter that is equal to the variance of the noise term e, and $w_{m}$ is a positive weight coefficient. The $w_{m}$ is defined as

(16) $$w_{m}=\left|\frac{\sigma_{y}\sigma_{g_{m}}}{Cov(y,g_{m})}\right|$$

where $\sigma_{y}$ and $\sigma_{g_{m}}$ are the standard deviations of vectors y and $g_{m}$, respectively; Cov(·) is the covariance function, and $g_{m}=[{a^{\prime }}_{m1}^{T},\;{a^{\prime }}_{m2}^{T},\;\ldots ,\;{a^{\prime }}_{mL}^{T}{]}^{T}\in {\mathbb{R}}^{LN}$ is a column vector composed of the *m*th atoms of $\Psi_{1}$, $\Psi_{2}$, …, $\Psi_{L}$. The value of $w_{m}$ is equal to the reciprocal of the correlation coefficient of y and $g_{m}$. It should be noted that large weights in the $\ell_{1}$-norm term of Equation (15) will encourage zero entries, while small weights will encourage nonzero entries according to the theory of compressed sensing [23]. The weights defined by Equation (16) can improve imaging performance with fewer artifacts, especially the “corner lighting” effect (artifacts with high magnitude appear at the corners of the interested imaging area) [14,24].

The problem of Equation (15) can be solved by many existing $\ell_{1}$-norm minimization algorithms, for example, a Matlab software tool box named SPGL1 [25]. Suppose that the solution of Equation (15) is $\hat{z}={\left[{\hat{z}}_{11},\;\ldots ,\;{\hat{z}}_{M_{2}1},{\hat{z}}_{12},\;\ldots ,\;{\hat{z}}_{M_{2}2},\ldots ,{\hat{z}}_{1L},\;\ldots ,\;{\hat{z}}_{M_{2}L}\right]}^{T}$, then the probability of damage in the *m*th grid under all L new scattering signals can be characterized as

(17) $$px_{m}={\left||\phi (m,\hat{z})|\right|}_{2}$$

In many Lamb wave imaging algorithms, such as delay-and-sum [8], minimum variance distortionless response [9] and dictionary-based imaging methods [14,24], usually the envelopes of the scattering signals instead of the original scattering signals are used because phase information contained in the scattering signals is difficult to be processed and predicted. It is found that imaging performance can be improved by replacing the original scattering signals with their corresponding envelopes [14]. Therefore, the corresponding envelopes are used to replace the original signal and atoms in Equation (15).

**Figure 2:**
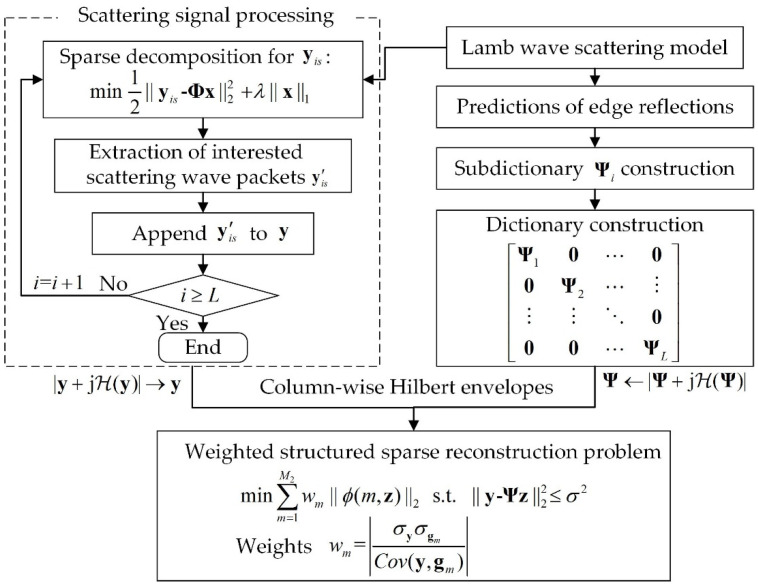
Schematic of the weighted structured sparse reconstruction-based Lamb wave imaging method.

(18) y←|y+jℋ(y)|, Ψ←|Ψ+jℋ(Ψ)|

where ℋ(·) is the column-wise Hilbert transform function. The schematic of the weighted structured sparse reconstruction-based Lamb wave imaging method is shown in Figure 2.

## 4. Simulation Validation

**Figure 3:**
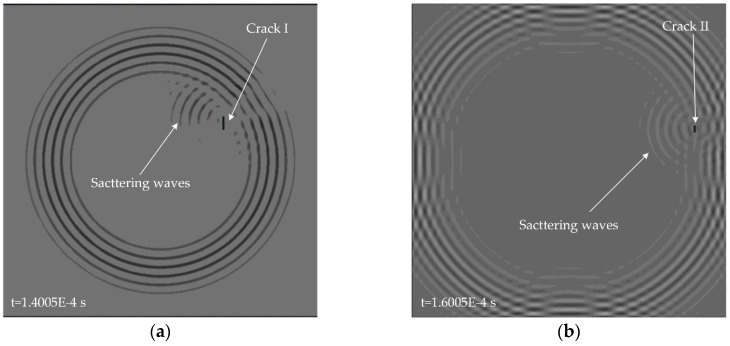
Snapshots of A0 mode Lamb wave propagation in an aluminum plate with different cracks: (**a**) crack I with a length of 20 mm and a width of 2 mm (simulation I); (**b**) crack II with a length of 10 mm and a width of 2 mm (simulation II). The excitation source is located on the center of the plate.

Simulations are implemented on an aluminum plate with the material properties listed in Table 1. The dimensions of the plate is 500 mm × 500 mm × 2 mm. A five-cycle Hanning-windowed tone burst centered at 80 kHz is excited on the center of the aluminum plate in a direction perpendicular to the surface of the plate. In this way, only A0 mode Lamb wave is excited. The wavelength of the A0 mode at the center frequency of 80 kHz is around 14.86 mm on the 2-mm aluminum plate. A square sensor array consisting of four measuring points is used to capture the response signals. Firstly, the baseline data is recorded on the intact plate without any defects. Then the scattering signals are obtained by subtracting the baseline from the response signals recorded after cracks introduced. Two different cracks with different lengths and locations are introduced in two separate simulations. Figure 3 shows the snapshots of the interactions between incident wave and cracks in the two separate simulations. It can be seen that the propagation of scattering waves is directional and the cracks designated are no longer ideal isotropic scattering source.

**Figure 4:**
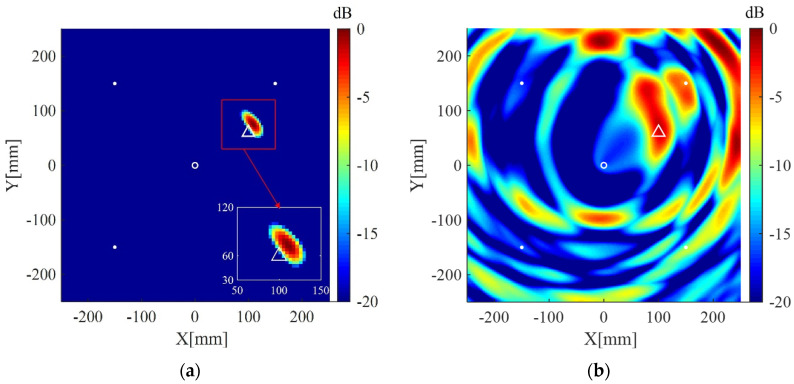
Imaging results in simulation I using: (**a**) the weighted structured sparse reconstruction-based Lamb wave imaging method (the location error is 17 mm); (**b**) the delay-and-sum imaging method (the location error is 195 mm). White dots denote locations of measuring points, the white circle denotes the location of excitation source, and the white triangle denotes the center of the crack I.

**Figure 5:**
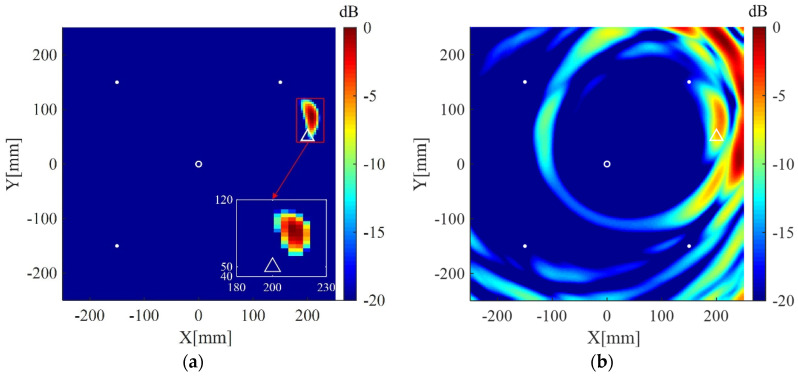
Imaging results in simulation II using: (**a**) the weighted structured sparse reconstruction-based Lamb wave imaging method (the location error is 34 mm); (**b**) the delay-and-sum imaging method (the location error is 179 mm). White dots denote locations of measuring points, the white circle denotes the location of excitation source, and the white triangle denotes the center of the crack II.

The plate is divided into 4 mm × 4 mm grids for imaging. The imaging results using the present method for the two cases are shown in Figure 4a and Figure 5a, respectively. The corresponding imaging results using the delay-and-sum method are also given in Figure 4b and Figure 5b. From the results one can see that although cracks do not act as isotropic scattering sources, the present method can still locate the cracks using data from the four measuring points. In addition, compared with the delay-and-sum method, the present method can obtain an image with few artifacts and smaller location error which is defined as the distance between the center of the crack and the location of maximum pixel value in the imaging result.

## 5. Experimental Validation

### 5.1. Experimental Setup

**Figure 6:**
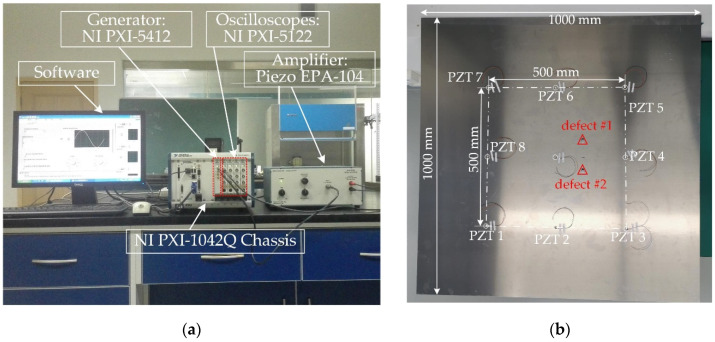
(**a**) Photograph of the experimental setup; (**b**) the experimental aluminum plate with PZTs.

Experiments are performed on an aluminum plate with the dimensions of 1000 mm length, 1000 mm width, and 2 mm thickness. The material properties of the experimental aluminum plate are the same as those used in the simulations. A step-pulse signal is generated by an NI PXI-5412 arbitrary waveform generator (National Instruments, Austin, USA), amplified to 60 V (peak-to-peak value) by a linear amplifier (Piezo EPA-104, Piezo Systems, Woburn, USA), and finally reaches to a Lead Zirconate Titanate (PZT) disk located on the center of the plate, which is 0.5 mm thick and 8 mm in diameter. The photograph of the experimental setup is shown in Figure 6a, and the experimental aluminum plate with PZTs and simulated defects is shown in Figure 6b. The resonant frequency $f_{r}$ of the PZT in thickness direction is 3.8 MHz and the frequency constant $N_{t}$ is 1900. Signals are received by eight PZTs with the same parameters as the exciting PZT and recorded by four NI PXI-5122 oscilloscopes(National Instruments, Austin, USA) with a sampling frequency $f_{s}$=10 MHz. Narrowband responses to a five-cycle Hanning-windowed tone burst centered at 80 kHz are recovered from the original step-pulse responses using the post-processing technique [26]

(19) $$U(\omega )=S(\omega )ℱ\{g^{\prime }(t)\}$$

where U(ω) is the desired narrowband response, ℱ{·} is the Fourier transform, g(t) is the step-pulse response and $g^{\prime }(t)$ is the first-order time derivative of g(t). The signal-to-noise ratio is increased through the above strategy of using the step-pulse excitation and post-processing technique [27]. The recovered narrowband responses to the tone burst centered at 80 kHz frequency are A0 mode dominant [28].

**Figure 7:**
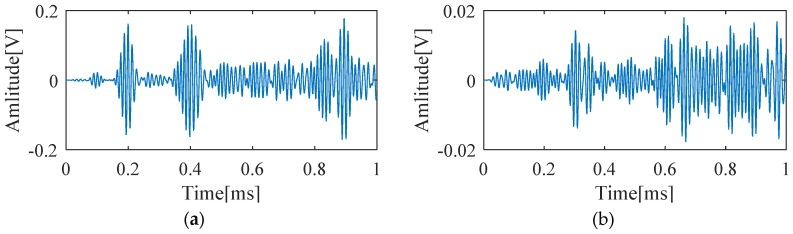
(**a**) The signal received by PZT 1 in the presence of defect #1; (**b**) the scattering signal obtained by subtracting the baseline from the signal shown in (a).

Cylindrical magnets with a diameter of 10 mm and a height of 10 mm are adsorbed on the same position of the opposite surfaces of the aluminum plate to simulate the scatterers with a uniform scattering feature. The magnets adsorbed on the plate act as an added mass loading that changes the boundary condition of the point. If the size of the cylindrical magnets is close to or larger than the wavelength of the incident wave, they can effectively scatter the incoming Lamb waves. Otherwise, the scattering waves caused by the cylindrical magnets will be very weak or even absent, and thus could not be recorded by the PZTs. In the experiment examination, the wavelength of the excited A0 mode is around 14.86 mm which is comparable to the diameter of the cylindrical magnets. Response signals are recorded first from the pristine plate, and scattering signals are obtained by subtracting the baseline signals from the corresponding ones recorded from the plate with added mass (cylindrical magnets). Figure 7a shows the received signal by PZT 1 after defect #1 is introduced, and the corresponding scattering signal is shown in Figure 7b. All scattering signals are finally down-sampled at a new sampling frequency, 1 MHz, to reduce data dimension.

### 5.2. Results and Discussion

**Figure 8:**
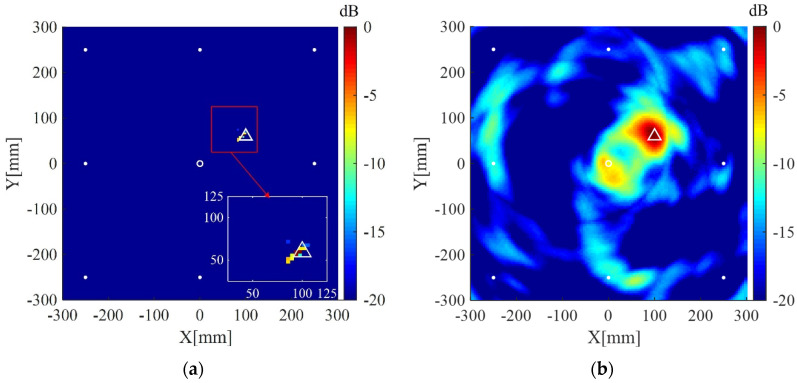
(**a**) Imaging result of the weighted structured sparse reconstruction-based Lamb wave imaging method using data from eight receivers (the location error is 3 mm); (**b**) Imaging result of the delay-and-sum imaging method using data from eight receivers (the location error is 3 mm). White dots denote locations of PZT receivers, the white circle denotes the location of excitation source, and the white triangle denotes the location of the scatterer.

**Figure 9:**
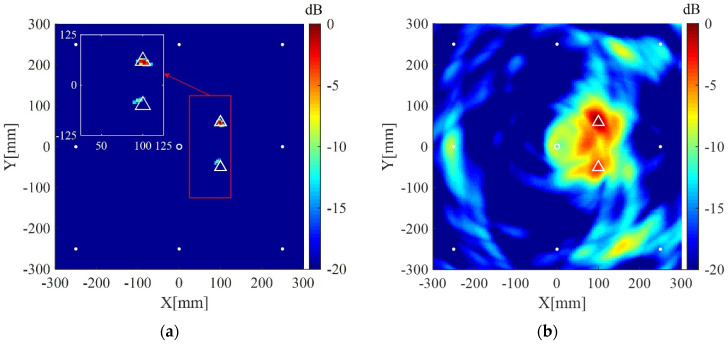
(**a**) Imaging result of the weighted structured sparse reconstruction-based Lamb wave imaging method using data from eight receivers for two scatterers (the location error is 3 mm for defect #1, and 8 mm for defect #2); (**b**) Imaging result of the delay-and-sum imaging method using data from eight receivers for two scatterers (the location error is 3 mm for defect #1, and 8 mm for defect #2). White dots denote locations of PZT receivers, the white circle denotes the location of excitation source, and the white triangles denote the locations of the scatterers.

The interested area is a square area with the dimensions of 600 mm × 600 mm located at the center of the plate, and is discretized to grids of 4 mm × 4 mm for imaging. The regularization parameters in Equations (9) and (15) are selected to be $\lambda =\lambda_{\max}/10=\max|\Phi^{T}y_{s}|/10$ and $\sigma^{2}=0.5{\left||y|\right|}_{2}^{2}$, respectively. The desired number of scattering wave packets $P^{\prime }$ in Equations (10) and (12) is selected to be 3. All imaging results are normalized to their corresponding maximum pixel values and displayed on a 20 dB color scale. For comparison, the corresponding results of the delay-and-sum imaging method are also given. Figure 8a shows the imaging result of the present method using data from the whole eight receivers. The scatterer is located in the image with a small spot size and without visible artifacts in 0 dB to −20 dB scale. The corresponding imaging result of the delay-and-sum imaging method is shown in Figure 8b, which exhibits some visible artifacts as well as a relatively larger spot size in the displayed scale. Figure 9a,b are the imaging results of the two methods for two scatterers, which are similar to the case of one scatterer.

**Figure 10:**
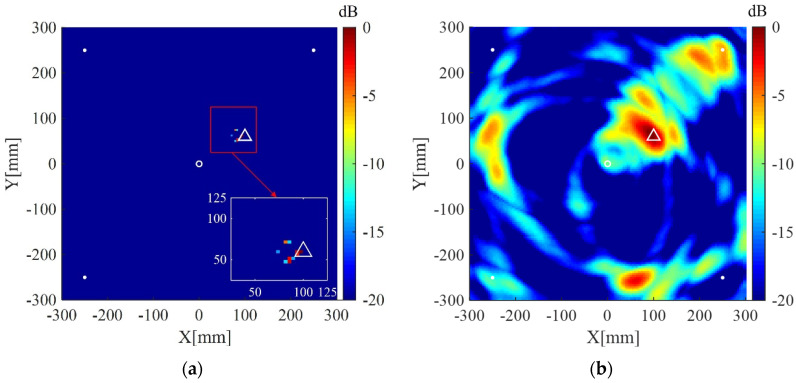
(**a**) Imaging result of the weighted structured sparse reconstruction-based Lamb wave imaging method using data from four receivers (the location error is 3 mm); (**b**) Imaging result of the delay-and-sum imaging method using data from four receivers (the location error is 3 mm). White dots denote locations of PZT receivers, the white circle denotes the location of excitation source, and the white triangle denotes the location of the scatterer.

**Figure 11:**
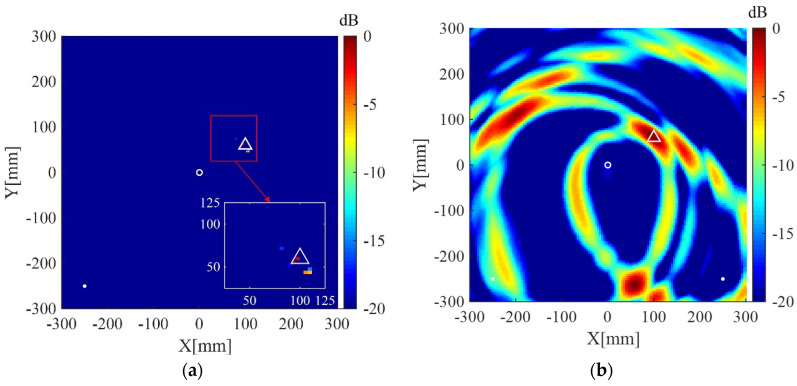
(**a**) Imaging result of the weighted structured sparse reconstruction-based Lamb wave imaging method using data from two receivers (the location error is 8 mm); (**b**) Imaging result of the delay-and-sum imaging method using data from two receivers (the location error is 324 mm). White dots denote locations of PZT receivers, the white circle denotes the location of the excitation source, and the white triangle denotes the location of the scatterer.

**Figure 12:**
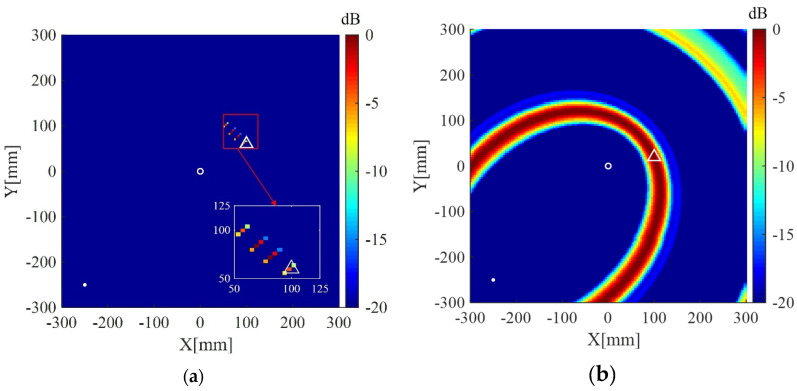
(**a**) Imaging result of the weighted structured sparse reconstruction-based Lamb wave imaging method using data from one receiver (the location error is 20 mm); (**b**) Imaging result of the delay-and-sum imaging method using data from one receiver (the scatter cannot be located). White dots denote locations of PZT receivers, the white circle denotes the location of the excitation source, and the white triangle denotes the location of the scatterer.

Imaging results of the present method and the delay-and-sum imaging method are also given in order to test the imaging performance of the two methods in the case of fewer receivers. As shown in Figure 10, Figure 11 and Figure 12, are the corresponding imaging results of the two methods using data from four receivers, two receivers and only one receiver, respectively. From the results, one can see that when the number of receivers is decreased, the performance of the delay-and-sum imaging method is decreased as the number of artifacts is increased. The imaging performance slightly decreases with the reduction in the number of the receivers for the present method. However, the change with the decrease in the number of the receivers is not so more obvious in the images of the present method compared with that of the delay-and-sum imaging method. The above results show that the delay-and-sum imaging method relies on the number of the receivers while the present method can achieve more sparse results with few artifacts.

**Figure 13:**
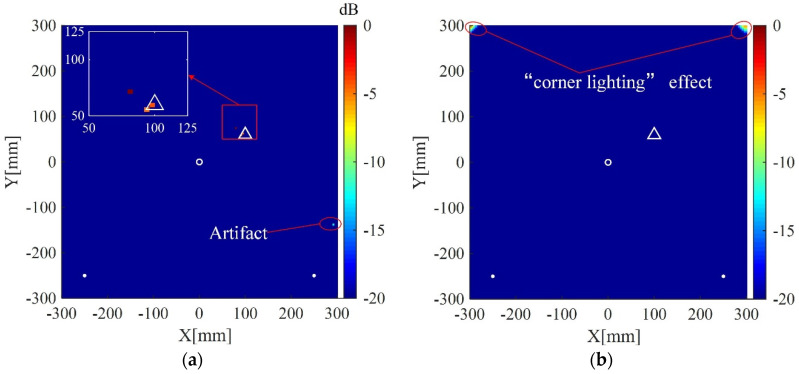
(**a**) Imaging result of the weighted structured sparse reconstruction-based Lamb wave imaging method using uniform weights (the location error is 20 mm); (**b**) Imaging result of the weighted structured sparse reconstruction-based Lamb wave imaging method using uniform weights and original scattering signals (the scatter cannot be located). White dots denote locations of PZT receivers, the white circle denotes the location of the excitation source, and the white triangle denotes the location of the scatterer.

**Figure 14:**
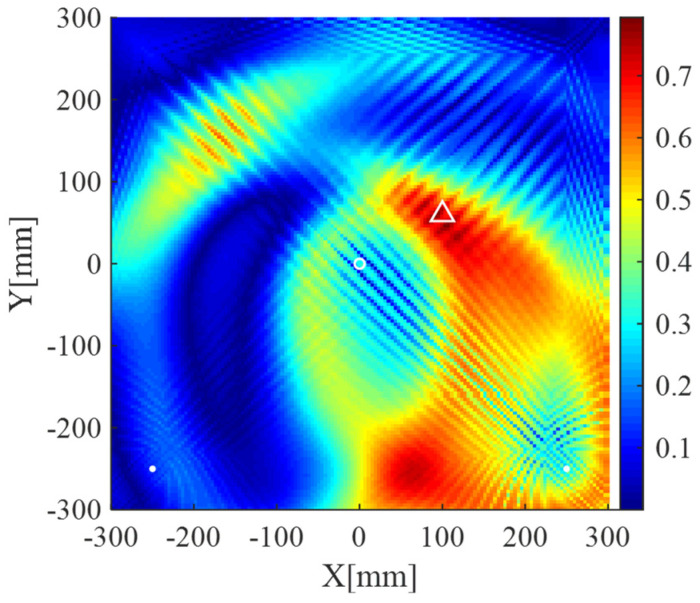
The images generated by the reciprocals of weights, i.e., $px_{m}=1/w_{m}$, using data from two receivers. White dots denote locations of PZT receivers, the white circle denotes the location of the excitation source, and the white triangle denotes the location of the scatterer.

Imaging result of the present method using uniform weights, i.e., the weights defined in Equation (16) is not used and replaced with $w_{m}=1\;(1\leq m\leq M_{2})$, is shown in Figure 13a. The result using uniform weights exhibits an artifact with small amplitude. In addition, Figure 13b shows the result of the present method using uniform weights and original scattering signals (signals without processed by Equations (9) and (10). In this case, the scattering signal processing operations described in Section 3.1 is not implemented. It can be seen from Figure 13b that the scatterer is not located correctly and the image exhibits the “corner lighting” effect. The corresponding reciprocals of weights are shown in Figure 14. As illustrated in Figure 14, the weights corresponding to the upper corners are relatively large, which encourage zero entries at those two areas (see the result in Figure 11a). On the contrary, the imaging methods using uniform weights cannot adaptively penalize the entries corresponding to the grids of the imaging area according to prior information, resulting in the degradation of imaging performance. Therefore, both the cases shown in Figure 13 verify the effectiveness of the weights defined in Equation (16) and the necessity of the scattering signal processing operation described in Section 3.1.

Lamb wave imaging method based on dictionary and unweighted sparse reconstruction can obtain results with smaller spot sizes because the $\ell_{1}$-norm optimization can lead to a sparse solution. However, it may not accurately locate the scatterers and even lead to the increase of artifacts when the atoms of the dictionary cannot precisely match scattering signal components (model mismatch) or an improper regularization parameter is used. The “corner lighting” effect can be suppressed in the present method because the defined weights corresponding to the corner area are relatively large (large weights will encourage zero entries in sparse reconstruction algorithms). Some of artifacts in the unweighted sparse reconstruction-based imaging method may also turn to be zero entries in the weighted one if the defined weights corresponding to those artifacts are large. More precise weights will lead to more accurate imaging results. However, it is hard to define the precise weights due to the unknown of the locations of scatterers. Fortunately, although precise weights are not available, the weights defined with the correlation coefficients between the atoms of the dictionary and the scattering signals can roughly locate scatterers. Therefore, imaging results with few artifacts and smaller spot sizes can be obtained using the weighted sparse reconstruction algorithm.

It is necessary to point out the limitations and requirements of the present method in the practical NDE. Because the present method is a $\ell_{1}$-norm optimization-based method, the computational complexity and storage is higher than the traditional delay-and-sum imaging method. In addition, the present method in current state aims at Lamb wave imaging for structures with regular shape, and it may not work for engineering structures with complex geometry because scattering waves and edge reflections in those structures are hard to be predicted

## 6. Conclusions

In this paper, a weighted structured sparse reconstruction-based Lamb wave imaging method is presented. Edge reflections are incorporated in the atoms of the dictionary, which is used to sparsely decompose the scattering signals. The Lamb wave imaging is formulated as a weighted structured sparse reconstruction problem. Several conclusions are drawn as follows:

1. Compared with the delay-and-sum imaging method, the present method can locate a scatterer using few receivers.
2. The imaging results of the present method exhibit few artifacts and smaller spot sizes compared with that of the delay-and-sum imaging method.
3. The defined weights in the present method can adaptively penalize the entries corresponding to the grids of the imaging area, which is helpful to alleviate the “corner lighting” effect and reduce artifacts.

However, the scattering coefficients of the edges are homogenized and still cannot accurately modeled, which produces errors in the prediction of edge reflections. In addition, more computation is needed to solve the weighted structured sparse reconstruction problem than that of the delay-and-sum method. Future work will focus on developing a more accurate prediction model for edge reflections.

## Figures and Tables

**Table 1:** Material properties of the experimental aluminum plate.

Young’s Modulus (*E*)	Poisson’s Ratio (ν)	Density (ρ)
68.9 Gpa	0.33	2690 kg/m^3^
